# Temperature tunability of surface plasmon enhanced Smith-Purcell terahertz radiation for semiconductor-based grating

**DOI:** 10.1038/s41598-017-06839-z

**Published:** 2017-07-25

**Authors:** Bo Han Cheng, Yu-Siou Ye, Yung-Chiang Lan, Din Ping Tsai

**Affiliations:** 1 Department of Photonics and Advanced Optoelectronic Technology Center, National Cheng Kung University, Tainan, 70101 Taiwan; 2 Research Center for Applied Sciences, Academia Sinica, Taipei, 115 Taiwan; 3 Department of Physics, National Taiwan University, Taipei, 10617 Taiwan

## Abstract

In this work, the terahertz (THz) Smith-Purcell radiations (SPRs) for the relativistic electron bunch passing over an indium antimonide (InSb)-based substrate with a subwavelength grating under various temperatures of substrate are investigated by FDTD simulations and theoretical analyses. The explored SPR is locked and enhanced at a certain emission wavelength with the emission angle still following the wavelength-angle relation of the traditional SPR. This wavelength agrees with the (vacuum) wavelength of surface plasmons (SPs) at the air-InSb interface excited by the electron bunch. The enhancement of SPR at this wavelength is attributed to the energy from electron concentrated in the excited SPs and then transformed into radiation via the SPR mechanism. When the temperature of InSb increases, the emission wavelength of the enhanced SPR decreases along with the emission angles increasing gradually. This work demonstrates that the emission wavelength and angle of the enhanced SPR from the InSb grating can be manipulated by the temperature of InSb. The temperature tunability of SP-enhanced SPR has potential applications in the fields of optical beam steering and metamaterial light source.

## Introduction

Strong demand of terahertz (THz) applications has attracted great attention in the development of compact and tunable light source. Especially, high-efficiency generation of THz light source is one of the most important issues and still remains to be overcome for the field of imaging and diagnostics^1, 2^. As an electron beam moves over a metallic surface, the surface plasmon (SP) on the surface can be excited. Subsequently, SP can be transformed into radiation modes by the designed structures such as slits, grooves and periodic gratings on the surface^3–6^. However, only the SPs whose operating frequencies close to the intrinsic plasma frequency of the metal can be efficiently excited. Hence the radiation frequencies are limited in optical and ultraviolet regions for most used noble metal^7^.

An electron beam passing over a metallic grating can also generate Smith-Purcell radiation (SPR). This type of radiation comes from the oscillation of charges and imaged charges on the grating which induces the periodically changing current density on the grating^8–12^. The emission frequency of SPR ranges from microwave to visible depending on the grating period and the electron velocity. Very recently, SPR that are manipulated by excitation of SPs and mimimic-SPs has been proposed and demonstrated^9, 10, 12–14^. Especially, ref. 10 investigates the condition that both the frequencies of SP and mimic-SP are out of the radiation band of SPR. The SPR is not enhanced and the radiations from SPR, SP and mimic-SP are all observed. References 13 and 14 propose and demonstrate amplification and manipulation of SPR by excitation of SPs on Ag film. And in refs 9 and 12, the generation of enhanced coherent THz SPR by excitation of mimic-SPs are proposed and explored.

The indium antimonide (InSb)–dielectric interface can also support propagation of SPs under illumination of terahertz light source^15^. The optical property of InSb at THz region is similar to that of noble metals in the optical region^16–18^. Furthermore, the optical properties of InSb can be tailored by changing its temperature^19^, by applying external magnetic field on it^20^ and by doping impurity into it^21^. Recently, many InSb-based tunable optoelectronic components based on controlling their temperatures have been studied, such as tunable photonic crystals^19^, infrared photo-detectors^22^, thermally controlled metamaterial^23–26^, data storage^27^, and subwavelength resolution^28^. Considering the above features of InSb, the enhanced and tunable THz light source can be implemented by applying the SPR with an InSb-based grating.

In this work, generating wavelength-adjustable narrow-band THz radiation via using SP-enhanced SPR on the InSb-based gratings and by controlling the temperature of substrate is proposed and investigated by FDTD simulations and theoretical analyses. This type of THz source with temperature-controlled emission wavelength and angle based on SP-enhanced SPR has never been proposed and investigated. The mechanism of concentration and enhancement of SPR at certain emission wavelengths and angles via excitation of SP is also elucidated by examining the effects of substrate temperature on the spectra of SP and SPR. The SPR on the InSb substrate provides a flexible device to verify the proposed SP-enhanced SPR mechanism. Here the manipulation of substrate temperature can be achieved by adding a heater under the substrate. In addition, part of emitted electrons may be absorbed in the substrate and hence increase the temperature of the investigated system^29^.

## Results

**Figure 1:**
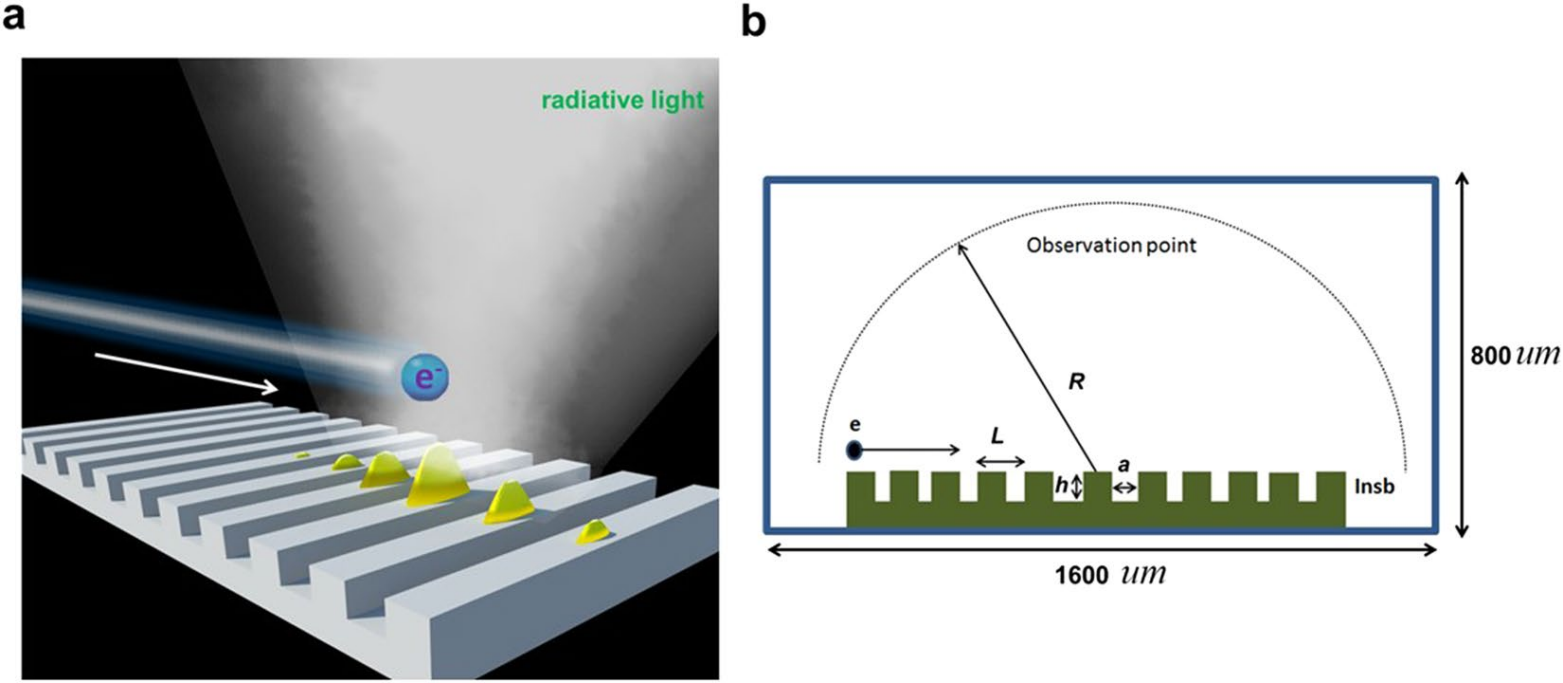
Schematic diagram and simulation model of proposed InSb-based grating for generating SP-enhanced SPR. (**a**) Schematic diagram. The yellow waves symbolize the excited SPs as electron bunch passes over the InSb grating. (**b**) Two-dimensional simulation model.

Figure 1a presents the schematic diagram of SPR emitting from a grating. Figure 1b plots the simulation model in this work. The grating is formed of an InSb substrate with carving ten periodic subwavelength grooves on it. An electron bunch moves above the proposed structure. In Fig. 1b, the period (*L*), width (*a*) and depth (*h*) of the groove are 45 *u*m, 22.5 *u*m and 22.5 *u*m, respectively. The distance (*R*) between the observation points and the center of the grating is 700 *u*m. (How the geometrical parameters affect the simulation results are analyzed in the section of Method and Materials.) The simulation domain occupies an area of 1600 × 800 *u*m^2^. The simulation method and detailed settings are given in the section of Method and Materials. The temperature-dependent dielectric constants of InSb are also shown in Method and Materials.

**Figure 2:**
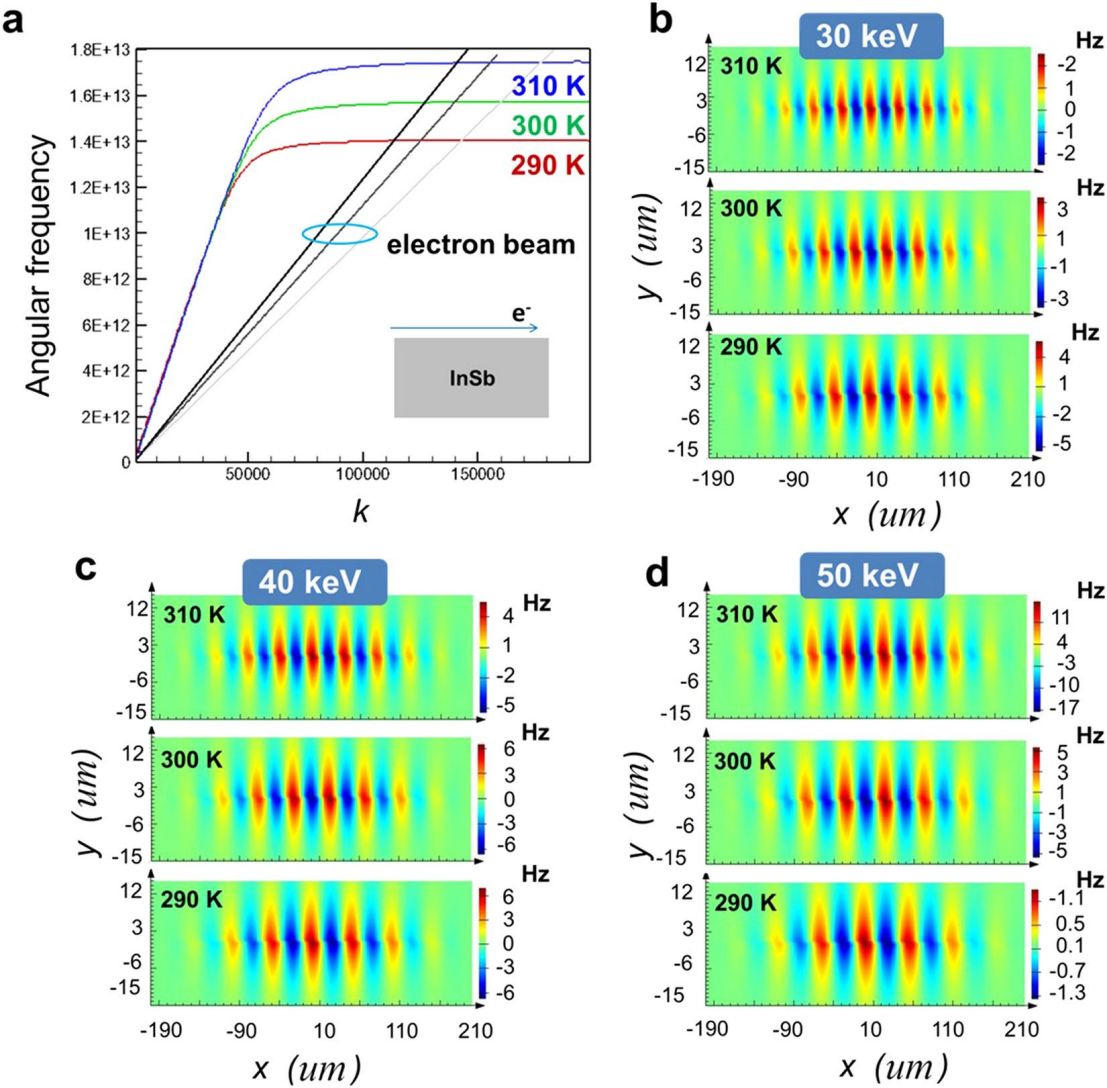
Dispersion curves and excited SPs at the air-InSb interface. (**a**) Blue (green, red) line: Dispersion curve of SP at the air-InSb interface with *T = *310 K (300 K, 290 K). Black line (dark grey, light gray): Dispersion curve of electron bunch with *E* = 50 keV (40 keV, 30 keV). (**b**), (**c**), and (**d**) Simulated Hz field contours of SPs excited by electron bunch passing over the InSb substrate under various temperatures (i.e., 290 K, 300 K, and 310 K) with *E* = 30 keV, 40 keV, and 50 keV, respectively.

To prove our concept, the behavior of SPs (excited by an electron bunch) propagating on the surface of InSb substrate is examined first. Figure 2a plots the dispersion curves of SPs at the air-InSb interface under various temperatures of InSb. The wavevector of excited SP (${{\rm{k}}}_{{\rm{sp}}}$) can be described as

1 $${k}_{sp}=\frac{\omega }{c}{(\frac{{\varepsilon }_{s}{\varepsilon }_{d}}{{\varepsilon }_{s}+{\varepsilon }_{d}})}^{1/2}$$

where *ω* is the angular frequency and *c* is the speed of light; ${\varepsilon }_{d}$ (${\varepsilon }_{s}$) denotes the relative permittivity of dielectric (the relative permittivity of InSb in THz region, see Method and Materials) (here ${\varepsilon }_{d}$ = 1). As the electron bunch passes over the InSb substrate, *ω* and ${k}_{sp}$ of the excited SP can be determined from the intersections of dispersion curves of SP and electron bunch as shown in Fig. 2a. Figure 2a reveals that the working points (*ω* and ${k}_{sp}$ of SP) change with the slope of dispersion curve of electron bunch, which is determined by ref. 30

2 $$\beta =\sqrt{1-\frac{1}{{\gamma }^{{\rm{2}}}}}=\frac{v}{c},$$

where *β* denotes the relativistic factor and *υ* and *c* are the speeds of electron and light, respectively; $\gamma =1+\frac{E\,(eV)}{0.511\times {10}^{6}\,eV}$ and *E* is energy of incident electron bunch. When the frequency of SPs at the air-InSb interface excited by electron bunch is within the emission frequency band of SPR, the intensity of SPR at this frequency will be enhanced (this phenomenon will be discussed later). Figure 2a also exhibits that increasing the energy of electron will decrease the wavevector of excited SPs (i.e. increase the wavelength of SPs). Furthermore, the frequency of excited SPs increases with temperature of InSb for a fixed electron energy, also shown in Fig. 2a. Hence, it is easier to observe and manipulate the SP-enhanced SPR by using the InSb substrate at THz region. Figure 2b,c and d plot the simulated contours of z-component magnetic field (Hz) for SPs at the air-InSb interface excited by the electron bunch with *E* = 30 keV, 40 keV and 50 keV, respectively, at temperatures (*T*) of 290 K, 300 K and 310 K. The calculated wavevectors of SPs in Fig. 2b–d and the correspondent frequencies agree with those in Fig. 2a well.

For an electron bunch passing over a metallic grating, the relation between the emission (vacuum) wavelength and angle of SPR can be express as^10, 31^

3 $${\lambda }=\frac{L}{|n|}({\beta }^{-1}-\,\sin \,\theta ),$$

where *λ* is the emission wavelength and *β* is the relativistic factor; *θ* denotes the observation angle measured from the direction normal to the grating surface (*θ* > 0(*θ* < 0) for forward (backward) emission); *L* is the period of grating and *n* denotes the order of SPR. Equation (3) originates from the constructive interference of radiation with emission wavelength λ emitted at an angle *θ* from two successive grooves of the grating initiated by the electron bunch passing above the grating with velocity ${\beta }c$. This SPR can also be viewed as the electron-excited SPR. Equation (3) reveals that the emission wavelengths strongly depend on the period of grating. For generating the light source in THz region, *L* is chosen as 45 *u*m in this study.

When SP on the substrate excited by electron bunch passes through the periodic gratings, its energy will be transformed into radiation emitted by the gratings. The wavevector-match condition for this process can be described as

4 $${k}_{sp}-n{k}_{G}=(\omega /c)\sin (\theta ),$$

where ${k}_{sp}$ denotes the wavevector of excited SP; ${k}_{G}=2\pi /L$ is the reciprocal wavevector of gratings; $\omega /c={k}_{light}$ is the wavevector of emission light, and n represents the diffraction order. Reorganizing Eq. (4) will yield Eq. (3) (i.e. SPR and this SP radiation have the same emission wavelength-angle relationship). Actually, the emission mechanisms of SPR and SP radiation are the same. Both of them originate from the constructive interference of radiation emitted from two successive grooves of the grating due to the oscillating current density on the grating. The only difference is that the oscillating current densities are induced by electron bunch in SPR and by SP in SP radiation. Therefore, SP radiation can be viewed as the SP-excited SPR. Furthermore, Eq. (4) also reveals that SP-excited SPR is angle-independent for different emission angle coming from diffraction order *n*. Besides, mimic-SP is also a kind of surface wave on the metallic surface with periodic structures and its property is similar to that of SP. The electron excited mimic-SP can also be transformed into radiation via periodic gratings. Hence, this process can also be viewed as the mimic SP-excited SPR.

**Figure 3:**
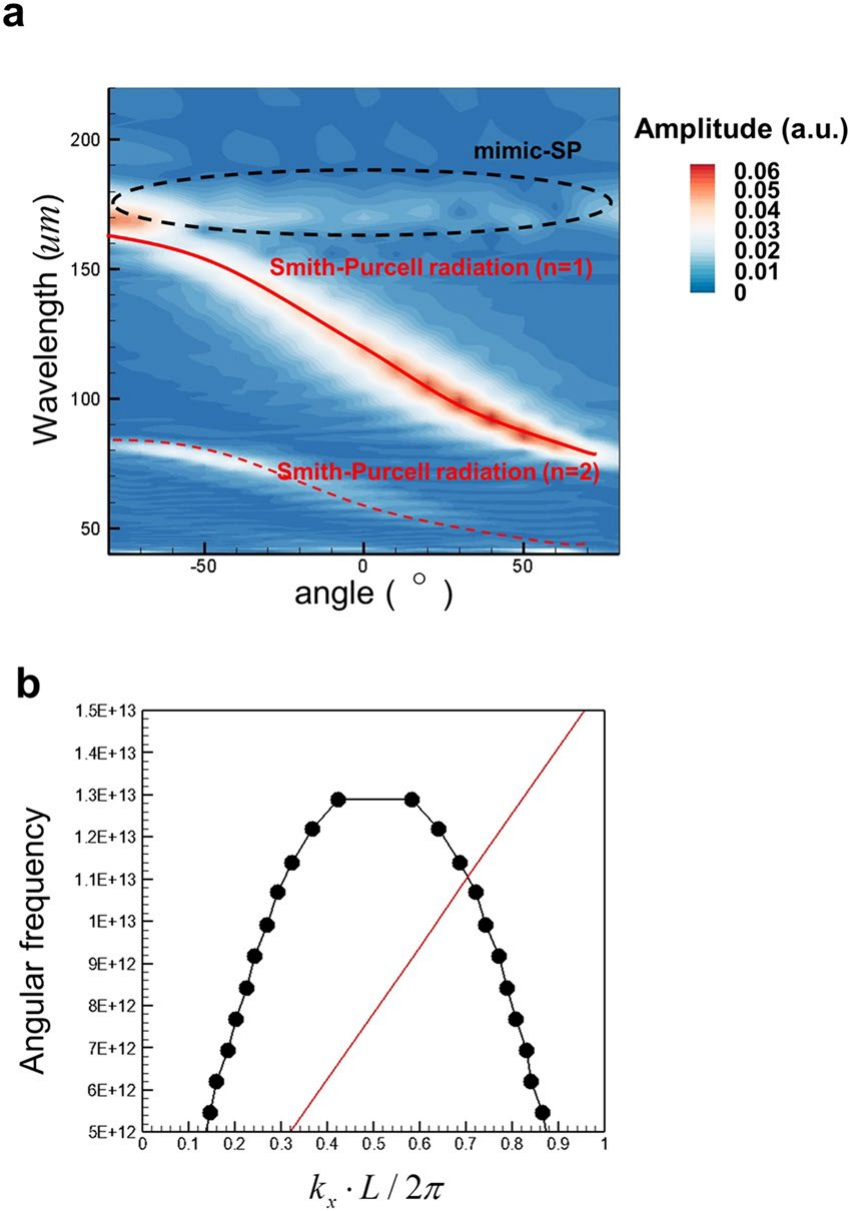
Simulated results for an electron bunch passing over a PEC grating. (**a**) Simulated contours of Fourier spectra of Hz fields as functions of emission wavelength and angle at the observation points. Red solid and dashed lines: wavelength-angle relation of traditional SPR for *n* = 1 and 2, respectively. (**b**) Dispersive curves of mimic-SP (black line) and electron bunch (red line). *E = *40 keV and *h* = 22.5 *u*m.

Next, the SPR from a perfect electric conductor (PEC) grating is investigated. It is considered as a standard SPR and the simulated results of SPR for an InSb grating will be compared with those of this standard SPR. Figure 3a presents the simulated contours of Fourier spectra of Hz fields as functions of emission wavelength and angle at the observation points (*R* = 700 um) for the radiation emitted from a PEC grating with *E* = 40 keV. As a comparison, the curves of wavelength versus angle for *n* = 1 and 2 of Eq. (3) are also plotted in Fig. 3a. Here the index *n* is the order of SPR (see Eq. (3)). It is the order of diffraction of the gratings. *n* = 1 and 2 correspond to the first and second, respectively, orders of SPR. Figure 3a indicates that the major part of the radiation is the first-order SPR which is a continuous spectrum with the wavelength between 75 *u*m and 165 *u*m and the angle from 90° to −90°. The second-order SPR is also observed in Fig. 3a but with the field amplitude being much smaller than that of the first-order SPR. Moreover, Fig. 3a also displays a weak radiation that is emitted in all direction with an unchanged wavelength. This type of radiation originates from the excitation of mimic-SP by electron bunch and then transformation of the mimic-SP into radiation by the grating. Figure 3b presents the dispersive curves of mimic-SP (obtained from FDTD simulation, see Method and Materials) and electron bunch with *E* = 40 keV. The intersection of these two dispersive curves gives the angular frequency (and its vacuum wavelength) of mimic-SP (here the angular frequency *ω* = 1.11 × 10^13^ *rad/s* and the vacuum wavelength *λ* = 170 *u*m), which confirms that the angle-independent radiation in Fig. 3a comes from the excitation of mimic-SP.

**Figure 4:**
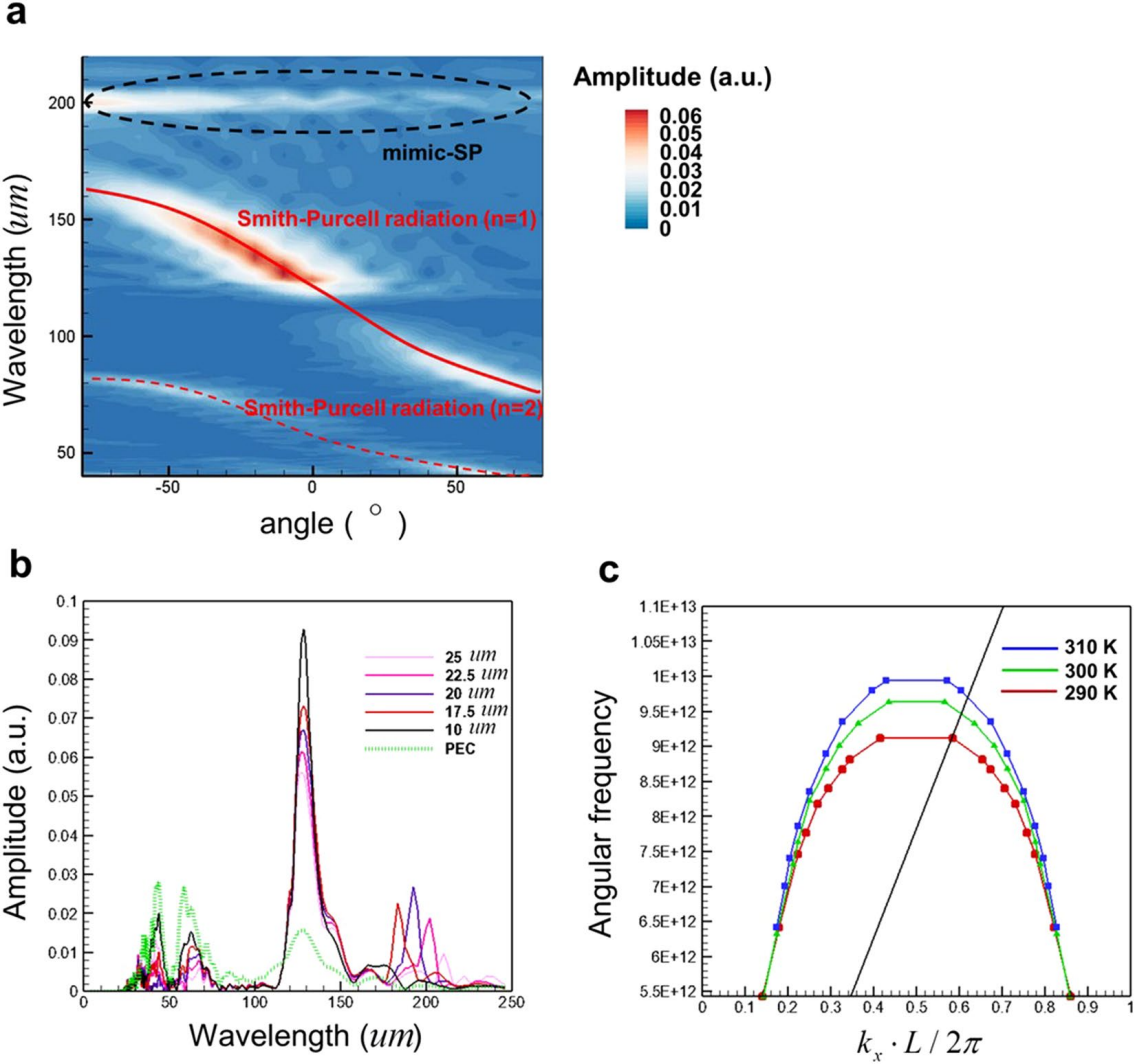
Simulated results for an electron bunch passing over an InSb grating. (**a**) Simulated contours of Fourier spectra of Hz fields versus emission wavelength and angle at the observation points (*h* = 22.5 *u*m). Red solid (dashed) line also denotes the wavelength-angle relation of traditional SPR for *n* = 1 (*n* = 2). (**b**) Simulated Fourier spectra of Hz field as a function of wavelength at *θ* = −10° (*R* = 700 *u*m) for an InSb grating with various values of *h* and a PEC grating with *h* = 22.5 *u*m. (**c**) Dispersive curves of mimic SP with various temperatures (red, green and blue lines) and electron bunch with *E* = 40 keV (black line). *h* = 22.5 *u*m.

Subsequently, the SPRs emitting from an InSb grating are explored. Figure 4a plots the simulated contours of Fourier spectra of Hz fields versus emission wavelength and angle at the observation points for the radiation emitted from an InSb grating with temperature *T* = 300 K and *E* = 40 keV. Here the wavelength-angle relations of the traditional SPR for *n* = 1 and 2 are also presented in Fig. 4a. Figure 4a shows that the relationship between wavelength and angle of the radiation still satisfies Eq. (3). However, the amount of radiation is concentrated at the wavelength *λ* = 120 *u*m (i.e. the angular frequency *ω* = 1.57 × 10^13^ *rad/s*) and *θ* = −10°. This specific wavelength (angular frequency) is consistent with that of the SPs at the air-InSb interface excited by electron bunch with *E* = 40 keV (see Fig. 2a, *T = *300 K). Compared to SPR from the PEC grating, the radiation intensities from the InSb grating are largely increased at *λ* = 120 *u*m but reduced at the other wavelengths of the SPR band. Figure 4b plots the Fourier spectra of Hz field as a function of wavelength at *θ* = −10° (*R* = 700 *u*m) for an InSb grating with various values of height (*h*) and a PEC grating with *h* = 22.5 *u*m. Figure 4b displays that at *λ* = 120 *u*m, the radiation from an InSb grating with *h* = 10 *u*m has the largest Hz-field amplitude and the amplitude decreases with the increase in *h*. And for *h* = 10 *u*m (*h* = 22.5 *u*m) at *λ* = 120 *u*m, the amplitude for an InSb grating is about six (four) times of that for a PEC grating.

Figure 4a and b imply that the SPR is enhanced at the specific emission wavelength and angle by excitation of SP. Changing the wavelength of the excited SPs within the SPR band will change the emission wavelength and angle of the enhanced SPR. The mechanism of SP-enhanced SPR is explained below. As the SP (mimic-SP) on the substrate is excited by electron bunch, the SP (mimic-SP) and electron have the same velocities. When the frequency of the electron excited SP (mimic-SP) is within the radiation band of electron-excited SPR, the radiations from electron-excited SPR and SP-excited SPR (mimic SP-excited SPR) are in phase and hence SPR is enhanced by the SP (mimic-SP). Conversely, if the frequency of the SP (mimic-SP) is out of the radiation band of SPR, the electron-excited SPR and SP-excited SPR (mimic SP-excited SPR) are two independent emission processes. In brief, the SP-enhanced SPR is attributed to that, when the frequency of SP is within the radiation band of SPR, the energy from electron concentrated in the excited SPs and then transformed into radiation via SPR mechanism. Notably, the SP-enhanced SPR can occur from THz to visible light strongly depending on the period of grating and plasma frequency of the substrate material. If InSb is replaced by the noble metal such as silver and gold, SP-enhanced SPR will move to visible light and infrared regions. (Of course, the grating period need to be reduced such that the radiation band of SPR is also moved to visible light and infrared regions.)

The angle-independent radiation from the excitation of mimic-SP with *λ* = 200 *u*m is also observed in Fig. 4a. To verify it, the dispersive curves of mimic-SP on the InSb grating are also examined. Figure 4c plots the dispersive curves of mimic-SP (also obtained from FDTD simulation) at *T* = 290, 300 and 310 K and electron energy of 40 keV. Figure 4c exhibits that at *T* = 300 K, the dispersion curves of mimic-SP and electron bunch intersect each other at *ω* = 9.42 × 10^12^ *rad/s* (i.e. *λ* = 200 *u*m), which confirms that the observed angle-independent radiation in Fig. 4a is ascribed to excitation of mimic-SP on the InSb grating. Moreover, Fig. 4c also indicates that the frequency (vacuum wavelength) of 40 keV-excited mimic-SP on the InSb grating increases (decreases) with the increase of *T*.

**Figure 5:**
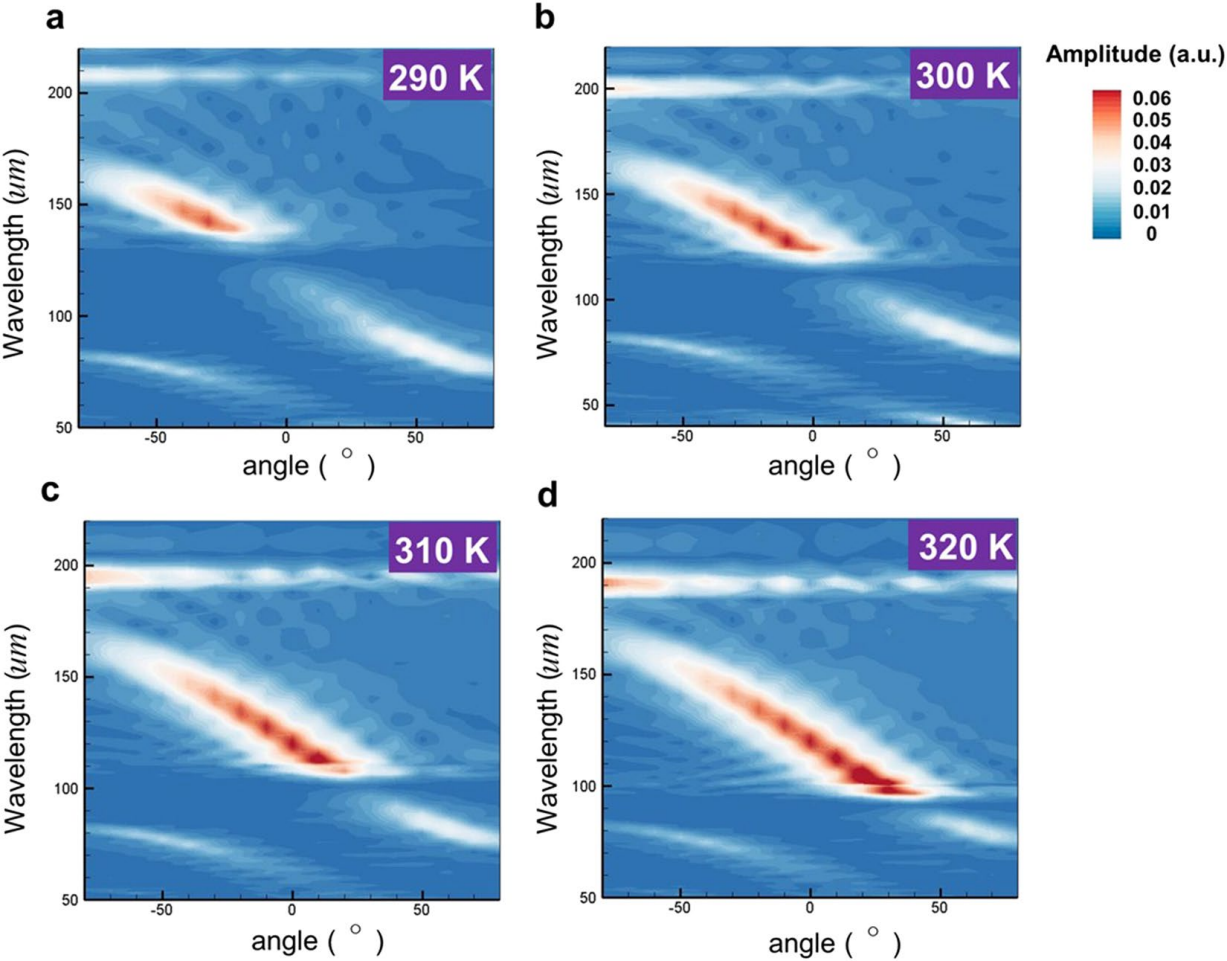
Simulated results for temperature effects on SP-enhanced SPR on an InSb grating. (**a**), (**b**), (**c**), and (**d**) Simulated contours of Fourier spectra of Hz fields versus emission wavelength and angle at the observation points with investigated temperatures of 290 K, 300 K, 310 K, and 320 K, respectively. *E = *40 keV and *h* = 22.5 *u*m.

Finally, the effects of InSb’s temperatures on the enhanced SPR are examined. The temperature will affect the carrier density (see Eq. (6)) and the plasma frequency in InSb. And the frequency of SP on InSb substrate excited by the electron bunch changes with InSb’s plasma frequency. Figure 5a,b,c and d present the simulated contours of Fourier spectra of Hz fields as functions of emission wavelength and angle at the observation points for the radiation emitted from an InSb grating with temperatures *T* = 290 K, 300 K, 310 K and 320 K, respectively, and *E* = 40 keV. (Note that, Fig. 4a is redrawn in Fig. 5b.) (Here these temperatures are chosen because we are only interested in the SP-enhanced SPR around room temperature and the temperature dependent carrier density formula (see Eq. (6), based on ref. 19) is only applicable to temperature between 260 K and 330 K.) Fig. 5a–d show that the emission wavelengths of the enhanced SPR decrease as the temperature increases along with the emission angles increasing from −30° to 30° gradually. The emission wavelengths of the enhanced SPR at various temperatures are in accordance with the vacuum wavelengths of excited SPs on the InSb grating at the correspondent temperatures as shown in Fig. 2a. The relationship between the emission wavelength of enhanced SPR and the emission angle for each *T* also follows the prediction of Eq. (3). In addition, Fig. 5a–d also exhibit that the emission wavelength of the angle-independent radiation owing to excitation of mimic-SPs decreases with the increase of temperature, which is consistent with the dispersion relations shown in Fig. 4c. These results demonstrate that the emission wavelength and angle of the enhanced SPR from an InSb grating can be manipulated by the temperature of InSb.

Figure 5a–d also exhibit a non-radiative band just below the emission wavelength of SP-enhanced SPR. For example, at *T* = 300 K (Fig. 5b), the wavelength of the non-radiative band is around 115 *u*m (its angular frequency is about 1.6  × 10^13^ *rad/s*, slightly larger than the frequency of the cross point, see the green line in Fig. 2a). This non-radiative band originates from SP on the InSb substrate with extremely large wavevectors also excited by the electron bunch. However, this kind of SP belongs to the so-called bonding mode (i.e. non-radiative mode). Its energy is strongly confined at the InSb-air interface and cannot be transformed into radiation. As a result, the emission spectrum displays a non-radiative band. Below the wavelength of non-radiative band, SP cannot be excited by the electron bunch any more. The observed weaker radiation in Fig. 5a–d comes from the original non-enhanced SPR. Because the plasma frequency and the SP frequency of InSb increase with the temperature of InSb, the wavelengths of SP-enhanced SPR and the non-radiative band decrease with the increase of temperature.

In our work, the structure and material parameters are designed such that the frequency of the SP (mimic-SP) is within (out of) the radiation band of electron excited SPR (*T* = 290 K ~ 320 K, *E* = 40 keV). Therefore, the strong SP-enhanced SPR and the weak angle-independent mimic SP-excited SPR are observed in Fig. 5a–d. Figure S1 of Supplementary Information presents the same simulated contours as those in Figs. 5a–d except for *T* = 270 K. At this temperature, the frequency of the SP is out of the radiation band of SPR. Therefore, both the electron-excited SPR (with a continuous emission band) and SP-excited SPR (which is angle independent and with a fixed wavelength) are observed with the peak field amplitudes being much smaller than those in Figs. 5a–d (i.e. at *T* = 270 K, the SPR is not enhanced by excited SP). These results further verify the proposed mechanism of SP-enhanced SPR and its temperature tunability.

This work also provides a possible solution to conduct an experiment for the proposed design. The InSb grating structure with various parameters can be fabricated on an InSb wafer by using the chemical vapor deposition and followed by the reactive ion etching to remove unwanted parts of InSb. The field emission gun of scanning electron microscope (SEM) can provide the electron beam. All of the components are mounted in a vacuum chamber. The emitted THz radiation at different positions can be collected by using THz fibers and delivered to a THz spectrometer^32^. The temperature tunability of the SP-enhanced SPR has potential applications in the fields of optical beam steering^33, 34^ and metamaterial light source^35^.

## Discussion

The SP-enhanced SPRs for an electron bunch passing over an InSb-based substrate with a subwavelength grating under various temperatures of substrate are investigated by FDTD simulations and theoretical analyses. The SPR is locked and enhanced at a certain emission wavelength with the emission angle still satisfying the wavelength-angle relation of the traditional SPR, Eq. (3). This wavelength agrees with the (vacuum) wavelength of SPs at the air-InSb interface excited by the electron bunch. The enhancement of SPR at this wavelength is attributed to the energy from electron concentrated in the excited SPs and then transformed into radiation via the SPR mechanism. When the temperature of InSb increases, the emission wavelength of the enhanced SPR decreases along with the emission angle increasing gradually. This work demonstrates that the emission wavelength and angle of the enhanced SPR from the InSb grating can be manipulated by the temperature of InSb. The temperature tunability of SP-enhanced SPR has potential applications in the fields of optical beam steering and metamaterial light source.

## Methods and Materials

Generally, in THz regime, the relative permittivity of InSb can be described as^16, 19^

5 $${\varepsilon }_{s}={\varepsilon }_{\infty }-{\omega }_{p}^{2}/({\omega }^{{\rm{2}}}+j\gamma \omega ),$$

where ${\varepsilon }_{s}$ and ${\varepsilon }_{\infty }$ are the frequency-dependent relative permittivity and high-frequency relative permittivity, respectively, of InSb; ${\omega }_{p}$ denote the plasma frequency of InSb; *ω* represents the angular frequency, and *γ* is the damping constant. In Eq. (5), ${{\omega }}_{{p}}$ is given by $\sqrt{N{e}^{{\rm{2}}}/{\varepsilon }_{{\rm{0}}}{m}^{\ast }}$ where *N*, *e*, ${{\rm{\varepsilon }}}_{{\rm{0}}}$ and *m* ^*^ denote the intrinsic carrier density, electron charge, free space permittivity, and effective mass, respectively. Comparing to the noble metal in visible regime, the plasma frequency of semiconductor is very sensitive to the temperature variation in THz regime (0.1–10 THz, i.e., 3000–30 *u*m). The relationship between carrier density (in unit of cm^−3^) and temperature can be expressed as^19^

6 $$N=5.76\times {10}^{20}{T}^{3/2}\exp (-0.26/2{{k}}_{{B}}{T}),$$

where *k*_***B***_ is the Boltzmann constant and *T* represents the temperature in Kelvin.

In this work, the FDTD based commercial electromagnetic software Lumerical is used in the simulation. The two-dimensional simulation is performed in the Cartesian x–y coordinate system. Here, the electron bunch is represented by a series of dipoles with phase delay that is related to the electron velocity. The dimensions of uniform grid cells in x and y directions are both set as 0.25 *u*m, which is enough for investigating both the radiative THz light and SPs^36^. The surrounding boundaries of the simulation model are perfectly matched layers (PMLs). Additionally, the simulation time is 40000 fs, which is long enough to record all the scattered signals from the grating at the measured positions.

To obtain the dispersion curves of mimic-SP of a grating by the FDTD method, we first put a line source (by setting the x-component electric field (Ex)) with a Gaussian temporal pulse in one groove of the grating and perform the simulation. Then, we take the temporal Fourier transform of the measured time-domain Ex field in another groove to acquire its frequency spectrum. The peak frequencies in the spectrum are the eigenfrequencies of the mimic-SP. Next, we use the same line source but with a sinusoidal temporal function of one eigenfrequency and perform the simulation again. Finally, the wavevector of this eigenfrequency is obtained by taking the spatial Fourier transform of the measured instant Ex field along the x direction on top surface of the grating. All the pairs of eigenfrequencies and the corresponding wavevectors constitute the dispersion curve of mimic-SP of the grating.

In this work, the period of groove (*L*) is chosen as 45 *u*m based on the fact that the frequency of SP on the InSb substrate around room temperature is within the radiation band of SPR to demonstrate the temperature tunability of SP-enhanced SPR. Hence, it cannot be chosen arbitrarily. The width of groove (*a*) is set as 22.5 *u*m. The width of groove does not affect the radiation band of SPR. Figures S2(a), (b), (c) of Supplementary Information present the same simulated contours as that in Fig. 5(b) except for the groove widths *a* = 15, 22.5 and 30 *u*m, respectively (*E* = 40 keV, *T* = 300 K). (Fig. S2(b) is the same as Fig. 5(b)). Figure S2(a–c) display that the emission wavelength and angle for SP-enhanced SPR are almost unchanged with the groove width. The groove width only affects the field amplitude of radiation. The depth of groove (*h*) is set as 22.5 *u*m. The effect of groove depth on the Fourier spectra of Hz field is discussed in Fig. 4(b). At *λ* = 120 *u*m and *θ* = −10° (the emission wavelength and angle of SP-enhanced SPR), the radiation for *h* = 10 *u*m has the largest Hz-field amplitude and the amplitude decreases with the increase in *h*. However, the groove depth does not affect the radiation band of SPR and the phenomenon of SP-enhanced SPR. Finally, the distance between observed points and gratings (*R*) needs to be large enough for measuring the far field of emission. In this work, *R* = 700 *u*m satisfies this requirement.

## Electronic supplementary material

Supplementary Information
