# Supplementary material for: Temperature tunability of surface plasmon enhanced Smith-Purcell terahertz radiation for semiconductor-based grating
Source: Sci Rep. 2017 Jul 25;7:6443. doi: 10.1038/s41598-017-06839-z (PMC5527105; doi:10.1038/s41598-017-06839-z)
Supplement: Supplementary file 1 — Supplementary Information [file 41598_2017_6839_MOESM1_ESM.pdf]

Supplementary Information

Temperature tunability of surface plasmon enhanced Smith-Purcell terahertz radiation for semiconductor-based grating

Bo Han Cheng, Yu-Siou Ye, Yung-Chiang Lan, and Din Ping Tsai

1. Figure S1

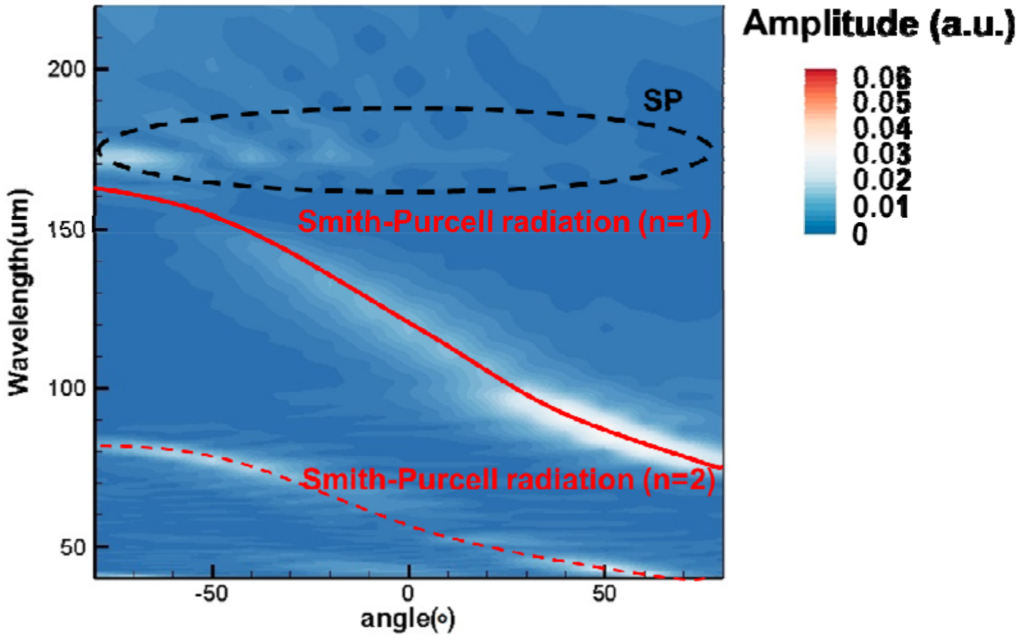

Fig. S1 Simulated contours of Fourier spectra of Hz fields versus emission wavelength and angle at the observation points with T = 270 K (E = 40 keV, a = 22.5 um and h = 22.5 um).

2. **Figure S2**

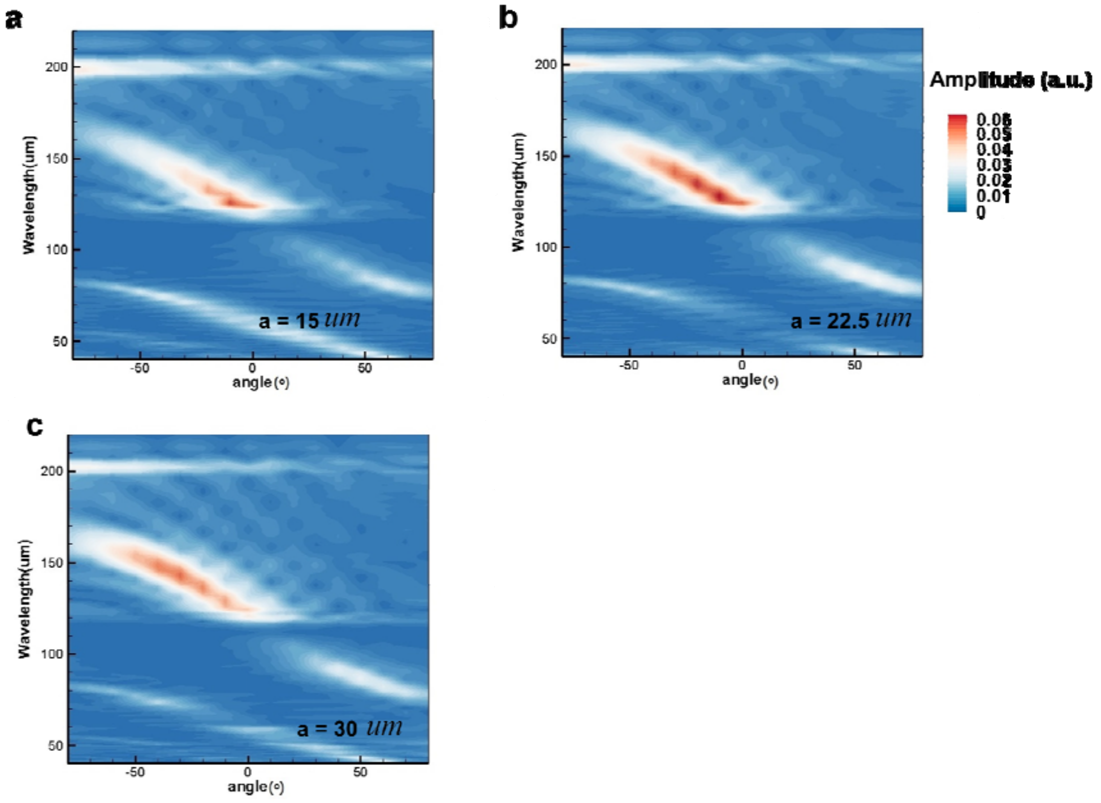

Fig. S2 (a), (b), (c) Simulated contours of Fourier spectra of Hz fields versus emission wavelength and angle at the observation points with groove widths *a* =15, 22.5 and 30 *u*m, respectively (*E* = 40 keV, *T* = 300 K and *h* = 22.5 *u*m).
